# kWIP: The *k*-mer weighted inner product, a *de novo* estimator of genetic similarity

**DOI:** 10.1371/journal.pcbi.1005727

**Published:** 2017-09-05

**Authors:** Kevin D. Murray, Christfried Webers, Cheng Soon Ong, Justin Borevitz, Norman Warthmann

**Affiliations:** 1 Research School of Biology, The Australian National University, Canberra, Australia; 2 Data61, CSIRO, Canberra, Australia; 3 Research School of Computer Science, The Australian National University, Canberra, Australia; UCSD, UNITED STATES

## Abstract

Modern genomics techniques generate overwhelming quantities of data. Extracting population genetic variation demands computationally efficient methods to determine genetic relatedness between individuals (or “samples”) in an unbiased manner, preferably *de novo*. Rapid estimation of genetic relatedness directly from sequencing data has the potential to overcome reference genome bias, and to verify that individuals belong to the correct genetic lineage before conclusions are drawn using mislabelled, or misidentified samples. We present the *k*-mer Weighted Inner Product (kWIP), an assembly-, and alignment-free estimator of genetic similarity. kWIP combines a probabilistic data structure with a novel metric, the weighted inner product (WIP), to efficiently calculate pairwise similarity between sequencing runs from their *k*-mer counts. It produces a distance matrix, which can then be further analysed and visualised. Our method does not require prior knowledge of the underlying genomes and applications include establishing sample identity and detecting mix-up, non-obvious genomic variation, and population structure. We show that kWIP can reconstruct the true relatedness between samples from simulated populations. By re-analysing several published datasets we show that our results are consistent with marker-based analyses. kWIP is written in C++, licensed under the GNU GPL, and is available from https://github.com/kdmurray91/kwip.

This is a *PLOS Computational Biology* Software paper.

## Introduction

A major application of DNA sequencing is comparing the genetic make-up of samples with one another to either identify commonalities, and thus detect relatedness, or to leverage the differences to elucidate function. Initially, one seeks to confirm assumed genetic lineages and replicates or to group samples into families, populations, and species. Estimating the genetic relatedness between a broad collection of samples must avoid bias and have minimal per sample cost.

Nowadays, the vast majority of studies in population genomics are performed using next generation sequencing (NGS) [1]. The methods commonly employed to analyse whole genome DNA sequencing data rely on two complementary concepts: the assembly of reference genomes and comparing samples to this reference by re-sequencing, read mapping, and variant calling. This approach, while functional in model organisms, is not ideal. Selecting the reference individual is mostly random, generating a reference genome assembly is time consuming and costly [2, 3], and analyses based on read alignment to a possibly inappropriate reference genome sequence are highly susceptible to bias [4, 5], to the point where large parts of the genomes are missed when sufficiently different or absent from the reference. Alignment-free methods for measuring genetic relatedness would help overcome this reference genome bias.

Another issue of concern is sample identification. A recent review [6] found that sample misidentification occurs at an alarming rate. With ever increasing sample numbers in (population) genetic projects, the issue of correct and consistent metadata arises on several levels: technical (mix-up) and biological (misidentification). Large field, and entire gene bank collections are being DNA-sequenced. With sample handling from the field through the laboratory to the sequence read files and eventual upload to data repositories, there is ample opportunity for mix-up and mislabelling of samples and files. This problem is exacerbated by the often highly collaborative nature of such undertakings. Some misidentifications, however, might be virtually undetectable without molecular genetic analysis, such as varying levels of ploidy, cryptic species, or sub-genomes in (compilo)species complexes [7]. Unfortunately, much of this hidden variation is easily overlooked by following aforementioned current best practices to calculate genome-wide genetic relatedness from short read sequencing data. Erroneous sample identification and/or underestimating the level of divergence has implications for downstream analysis choices, such as which samples and populations to use for a Genome Wide Association Study (GWAS); the missing heritability might then in fact be in the metadata.

The field of alignment-free sequence comparison aims to combat these difficulties by avoiding the process of sequence alignment. Approaches include decomposition into words, i.e., substrings of length k, commonly referred to as *k*-mers [8–11], sub-string or text processing algorithms [12–14], and information theoretic measures of sequence similarity or complexity [15]. While avoiding sequence alignment, some alignment-free sequence comparison tools still require prior knowledge of the underlying genome sequences, which precludes their use as a *de novo* tool. Recently, several algorithms enabling *de novo* comparisons have been published. These extensions all attempt to reconstruct phylogenetic relationships from sequencing reads. Spaced [13, 16] uses the Jensen-Shannon distance on spaced seeds (small *k*-mers a short distance from one another or with interspersed disregarded bases) to improve performance of phylogenetic reconstruction. Cnidaria [17] and AAF [18] use the Jaccard distance to reconstruct phylogenies, while mash [19] uses a MinHash approximation of Jaccard distance to the same effect.

One of the most established and studied alignment-free sequence comparison metrics is the *D*_2_ statistic [8, 10]. It measures the difference between two sequences by the number of *k*-mer matches. First, all *k*-mers are counted in each sequence and recorded in a count vector. Then the difference between those vectors is measured. In the case of the original *D*_2_ statistic, this is achieved by simply building the vector product. Several derivatives of the *D*_2_ statistics, e.g., $D_{2}^{*}$, $D_{2}^{S}$, have been developed over the years [8, 20–23], which aim to improve accuracy by modelling and correlating observed versus expected *k*-mer frequencies. While these statistics have been extended to Next Generation Sequence data [24] and successfully applied to metagenome comparisons [25], these *D*_2_ statistic derivatives, such as $D_{2}^{*}$ and $D_{2}^{S}$, have the significant drawbacks of slow computational speed and the difficulties of defining the background models.

Here we present the *k*-mer Weighted Inner Product, a new metric to estimate genetic relatedness that introduces and combines two concepts to *k*-mer-based sequence comparison. Similar to the *D*_2_ statistic(s), the similarity measure is an inner product of *k*-mer counts, but firstly, we no longer compare every *k*-mer, but rather hash all *k*-mers of a sample into a probabilistic data structure: a sketch [26]. The resulting sketches are, in effect, vectors of *k*-mer counts; importantly, the sketches for all samples have a constant size. Secondly, we introduce an information-theoretic weighting to elevate the relevant genetic signal above the noise. Pairwise similarity is then calculated by the inner product between *k*-mer counts, weighted by the information content derived from their frequencies across the population. Our procedure is implemented in a software tool (kWIP) that calculates our metric, the *k*-mer Weighted Inner Product, directly from sequencing reads. We show by simulations and by re-analysing published datasets, that kWIP can quickly, and accurately detect genetic relatedness between samples.

## Design and implementation

**Fig 1:**
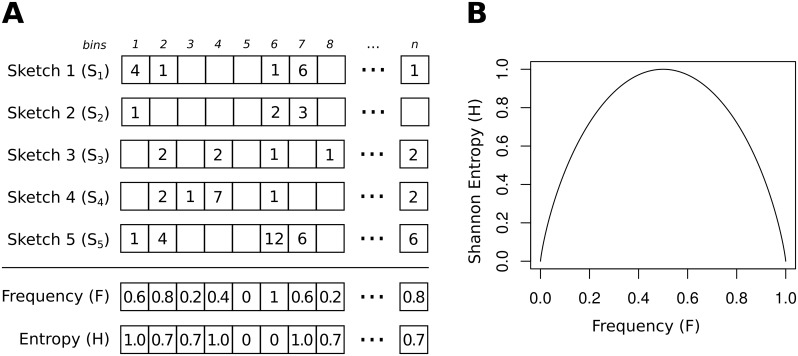
Overview of the weighted inner product metric as implemented in kWIP. (A) *k*-mers are counted into sketches (using khmer [28]). Columns represent the “bins” in each sketch. The frequencies of non-zero counts across a set of sketches is computed, forming the population frequency sketch (denoted *F*). We calculate Shannon entropy of this frequency sketch as the weight vector for the WIP metric (denoted *H*, see Eq 2). (B) Illustration of Shannon Entropy as used in kWIP: the relationship between the population frequency (*F*) and the weight (*H*).

kWIP operates on files containing sequencing reads generated by common modern sequencing platforms (e.g., Illumina). First, kWIP utilises khmer [27, 28] to count overlapping words of length *k* (*k*-mers) into a probabilistic data structure, a sketch, for each sample. In order to establish the weights kWIP then counts presence/absence of each *k*-mer across all sample sketches and records this population occurrence frequency in a frequency sketch (*F*). We calculate similarity (*K*) as the inner product between each pair of sample sketches, weighted by the Shannon entropy (*H*) of the respective frequency (*F*). The concept is illustrated in Fig 1.

### *k*-mer counting

For each sample, kWIP uses khmer to decompose sequencing reads into overlapping words of some fixed length k, e.g., 20. The value of a reversible hash function is computed for each *k*-mer. *k*-mers are canonicalised by using the lexicographically smaller of a *k*-mer and its reverse complement. *k*-mers are counted using one sketch per sample. These sketches are vectors with prime number length, typically several billion elements in size (denoted *S*_*i*_ for sample *i*). The elements of these sketches are referred to as bins (indexed by *b*, e.g. $S_{i_{b}}$), and can store values between 0 and 255 (integer overflow is prevented). To count a *k*-mer, the *b*-th bin of the sketch ($S_{i_{b}}$) is incremented, where *b* is the hash value of the *k*-mer modulo the (prime) length of the sketch. For most use cases, *k*-mers between 19 and 21 bases long should achieve a good balance between specificity and sensitivity across genomes and genomic regions [29]. Note that the possible number of *k*-mers (4^k^) is much larger than the length of a sketch. Therefore, aliasing (or “collisions”) between *k*-mers can occur, but in practice can be avoided with appropriate parameter selection [27]. It is worth noting that aliasing can only increase similarity between any two samples and should occur uniformly across all sample pairs.

### Weighting and similarity estimation

Genetic similarity is estimated by calculating the inner product between each pair of sample sketches (*S*_*i*_, *S*_*j*_), weighted by the informational content of each bin. The population frequency sketch (*F*) contains the frequency of occurrence for each bin, calculated as the proportion of samples with a non-zero count for each bin. We calculate a weight vector (*H*) of these occurrence frequencies using Shannon entropy as per Eq (1). In the Weighted Inner Product (WIP) metric (or kernel), pairwise similarities are then calculated as the inner product over every pair of sample sketches, weighted by *H* as per Eq (2). The unweighted Inner Product (IP) metric is simply the inner product between the two sketch vectors, $S_{i}^{T}S_{j}$, without weighting. This produces a matrix of pairwise inner products *K*, commonly referred to as a kernel matrix. The kernel matrix is then normalised using the Euclidean norm Eq (3), and converted to distances using the “kernel trick” [30] as per Eq (4). To ensure distance matrices are Euclidean, kWIP confirms that the resulting kernel matrix is positive semi-definite by checking that all eigenvalues are non-negative using the Eigen3 library [31].

The distances kWIP produces are relative within the set of samples being compared. This is because the weight vector (*H*) is specific to the set of samples and the similarity estimates are normalised to account for varying sequencing coverage. In other words, the kWIP distance for a given pair of samples will depend on the set of samples within which they are analysed.

$$\begin{array}{c} H=-(F\log_{2}\left(F\right)+\left(1-F\right)\log_{2}\left(1-F\right)) \end{array}$$ (1)

$$\begin{array}{c} K_{ij}=\sum_{b=1}^{n}S_{i_{b}}S_{j_{b}}H_{b} \end{array}$$ (2)

$$\begin{array}{c} K_{ij}^{\prime }=\frac{K_{ij}}{\sqrt{K_{ii}K_{jj}}} \end{array}$$ (3)

$$\begin{array}{c} D_{ij}=\sqrt{K_{ii}^{\prime }+K_{jj}^{\prime }-2K_{ij}^{\prime }} \end{array}$$ (4)

### Implementation

Pairwise calculation of genetic distances from *k*-mer count files with both the WIP and IP metrics is implemented in C++ as kWIP. kWIP is licensed under the GNU GPL, and source code and pre-compiled executables are available from https://github.com/kdmurray91/kwip. Documentation and tutorials are available from https://kwip.readthedocs.io. To use kWIP, one first counts *k*-mers present in each sample using khmer’s load-into-counting.py script [28]. kWIP will then estimate similarity from these counts, producing a normalised Euclidean distance matrix and, optionally, the corresponding similarity matrix (kernel matrix). kWIP parallelises pairwise similarity calculations across cores of a multi-threaded computer to ensure fast operation.

## Results

We show that kWIP is able to accurately determine genetic relatedness in many scenarios. Using a simulated population re-sequencing experiment, we quantify how the population frequency-based weighting applied by kWIP improves accuracy, that is the correlation with the known truth, when compared to existing approaches, mash [19], and the unweighted metric, IP. We recover known technical and biological relationships between sequencing runs of the 3000 Rice Genomes project [32, 33]. We show that kWIP’s estimate of genetic relationships between *Chlamydomonas* samples is nearly identical to results obtained by a more traditional, SNP-based analysis employing read mapping and variant calling against a reference genome with the same sequencing data [34]. By analysing a dataset on root-associated microbiomes [35], we show that our approach of sample clustering by kWIP can be extended to clustering of metagenome samples.

### Quantification of kWIP performance

**Fig 2:**
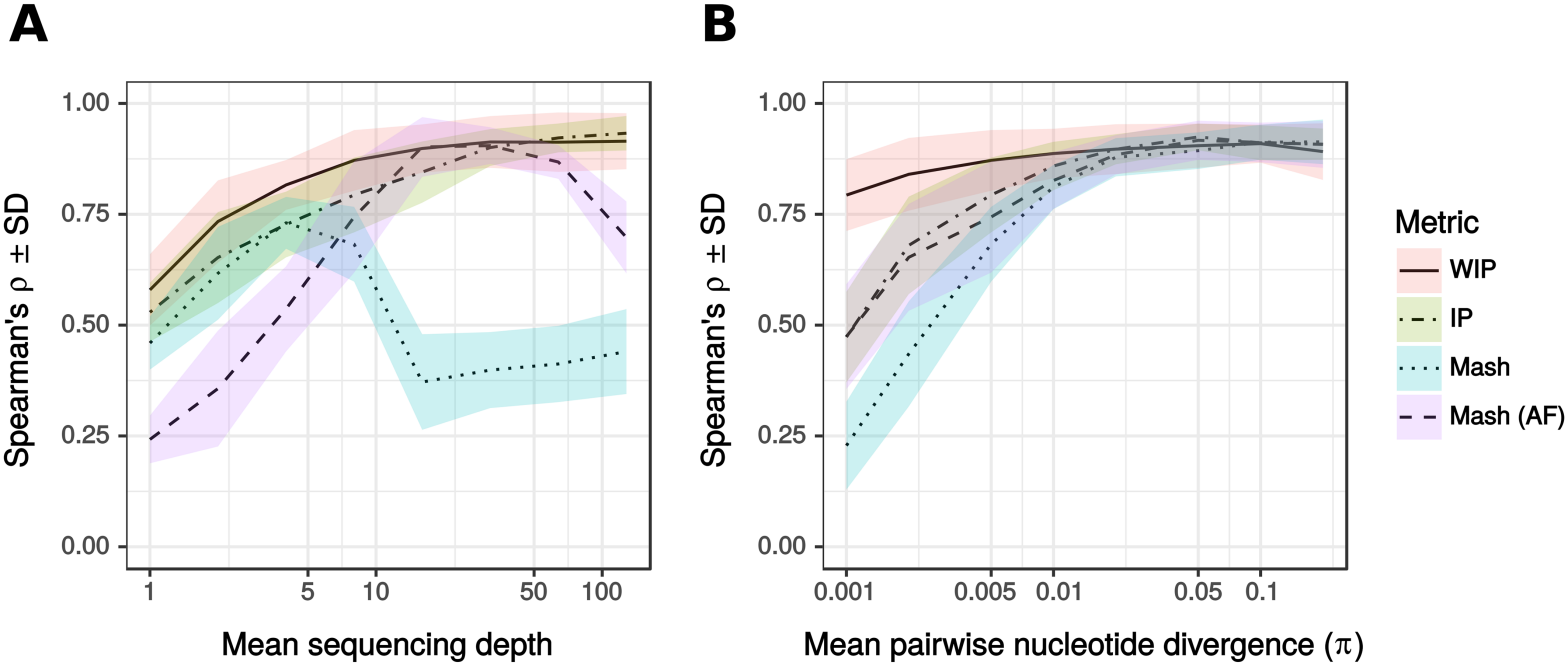
The effect of (A) mean sequencing depth (genome coverage) and (B) average number of nucleotide differences per site (*π*) on accuracy of genetic similarity estimates in simulations. We plot mean ± standard deviation of Spearman’s *ρ* comparing each metric to known truth across 20 replicate runs. (A) Mean sequencing depth varies while average number of nucleotide differences per site (*π*) is constant at 0.005. kWIP: At low to moderate mean sequencing depth (<30x) weighting increases accuracy. The weighted metric (“WIP”) obtains near-optimal accuracy already at 10x and hence much earlier than the unweighted metric “IP”). There is no noticeable decrease in accuracy with increasing coverage. mash: regardless of error correction, mash performs less well than WIP. mash shows accuracy maxima at 4x coverage without (“Mash”) and at 16x coverage with abundance filter (“Mash (AF)”), at which point Mash (AF) performs almost as well as WIP. The accuracy of mash decreases dramatically when coverage is further increased. (B) Genome coverage is kept constant at 8x and average number of nucleotide differences per site (*π*) varies. While all metrics perform equally at a (*π*) of 1 in 100 (0.01), the performance of IP, Mash and Mash (AF) decreases rapidly as (*π*) between samples decreases. This does not occur for the weighted metric (WIP).

We quantified the performance of kWIP with simulated population sequencing data. We compare our novel metric, the weighted inner product (WIP), to the unweighted inner product (IP), which we consider equivalent to the *D*_2_ statistic, and to mash [19]. We simulated 20 populations of 12 individuals with 1 MBp genomes and analysed each with kWIP and mash for *k*-mers of k = 20. A summary of the results of these 20 replicate analyses with each of the metrics is shown in Fig 2.

Unsurprisingly, for all metrics the accuracy, that is the rank correlation (Spearman’s *ρ*) to known truth, decreases with decreasing genome coverage, i.e., average sample sequencing depth (Fig 2A), as well as with decreasing average number of nucleotide differences per site, *π* (Fig 2B).

Importantly, at low coverages, the weighted metric (“WIP”) performs better than the unweighted (“IP”) (Fig 2A). Above a certain coverage, in the case of our simulations above about 30-fold, the performances of the WIP and IP metrics converge. At a constant genome coverage, the improvement in accuracy of the WIP metric relative to the IP metric increases as mean pairwise genetic variation decreases (Fig 2B). While the accuracy of the IP metric decreases markedly below an average number of nucleotide differences per site (*π*) of approximately 0.01, the WIP metric does not show such decrease.

In order to compare the performance of kWIP relative to Mash [19] we conducted two analyses with mash: one with abundance filtering enabled to remove singleton *k*-mers (“Mash AF”) and one without (“Mash“). Within the scope of our simulations kWIP yields more accurate results than mash when sequencing coverage and/or sequence divergence is low; a typical scenario in large-scale, population genetic analyses within species. Through the entire range of simulation parameters, kWIP never yields results less accurate than mash, irrespective of abundance filtering (Fig 2). It is interesting to note that mash appears to exhibit characteristic accuracy maxima, and accuracy decreases dramatically when mean sequencing depth is further increased. In addition, abundance filtering seems to have a strong, genome coverage-dependent effect on the accuracy of mash (Fig 2A). With the chosen parameter settings, mash runs much faster than kWIP (about 10-fold faster; see performance comparisons in Table 1).

**Table 1:** Computational performance of kWIP.

Dataset	Dataset Size			Distance Calculation Time (s)		
	Samples	Reads	*k*-mers	Mash	WIP	IP
Simulation (8x)	36	7.9e4 ± 2.4e3	1.3e6 ± 1.3e5	6 ± 1	45 ± 3	40 ± 4
Simulation (32x)	36	3.2e5 ± 1.0e4	2.3e6 ± 3.8e5	5 ± 1	53 ± 3	46 ± 5
Rice Replicates	96	9.7e6 ± 1.5e6	1.8e8 ± 1.5e7	-	2241 ± 139	1892 ± 286
Chlamydomonas	20	2.0e8 ± 2.4e7	1.4e8 ± 1.4e7	-	127	194

Measurements of calculation time are in wall-clock seconds on a 16-core, 64GB GNU/Linux server. Figures are means ± standard deviations. For simulations, these are over the 20 replicate runs performed. For rice replicate clustering, these are over 10 of the 100 independent sets of 96 rice samples. For Chlamydomonas, times are for the full dataset. The sketch sizes used were 10^9^ bins for kWIP/khmer, and 10^4^ for Mash. Note that *k*-mers refers to the number of distinct *k*-mers as estimated by khmer.

In analyses with kWIP we find that the coefficient of variation between the number of sequencing reads per sample matters. For samples with much lower mean sequencing depth than the average, kWIP has difficulty to accurately determine its relatedness to other samples. We therefore advise to exclude such samples from kWIP analyses or sub-sample reads from the remainder, if the dataset allows. khmer provides procedures for “digital normalisation”, which can be used upstream of kWIP to that effect [36]. Our simulations suggest that variations in genome coverage between samples will also affect the results obtained with mash.

### Replicate clustering

**Fig 3:**
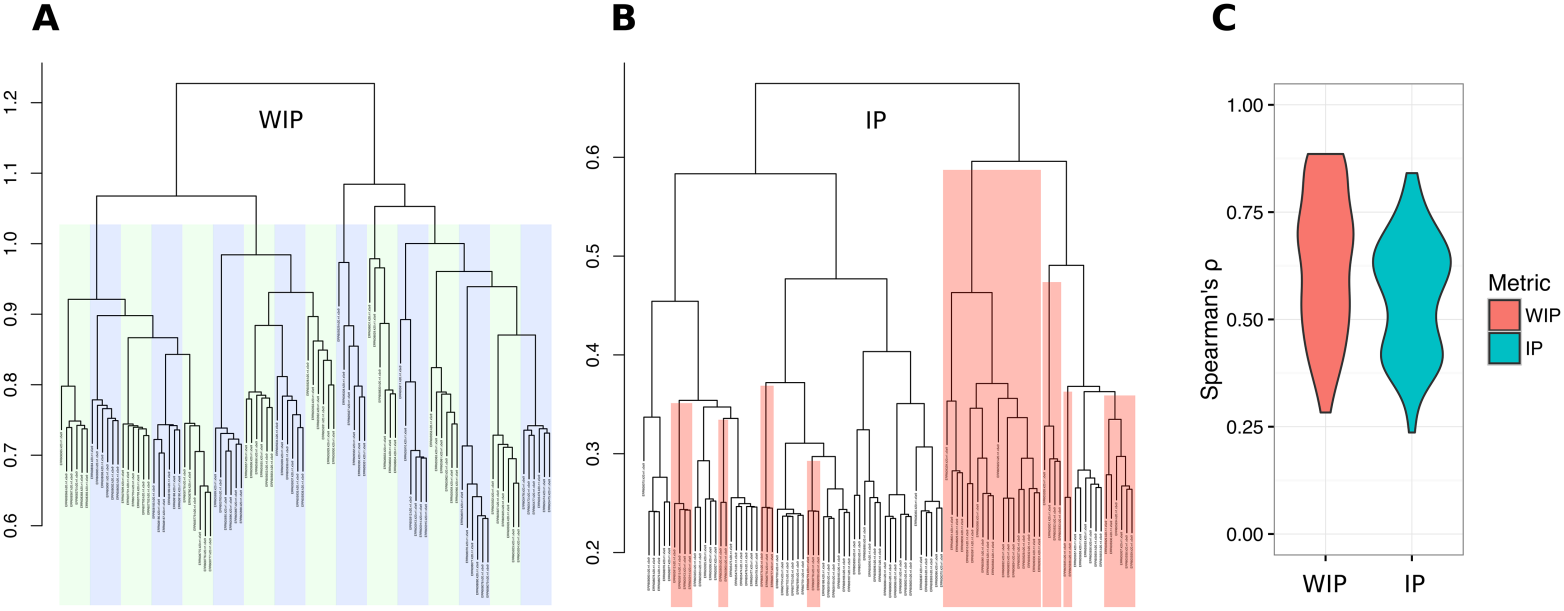
Weighting improves the accuracy of replicate clustering. (A) and (B) show a representative example, demonstrating that (A) the weighted metric (WIP) correctly clusters all sets of 6 replicate runs into their respective samples (indicated by blue and green bars) while (B) the unweighted metric (IP) fails to cluster several replicates correctly (indicated by red highlighting). (C) rank correlation coefficients to expected relationships over 100 sets of 96 rice runs for the WIP and IP metrics. The Weighted metric tends to cluster the replicates better.

kWIP can efficiently verify replicates. Fig 3A and 3B show a representative example of replicate clustering. The weighted metric (WIP) is able to accurately cluster replicates (Fig 3A), whereas the unweighted metric (IP) makes mistakes, as highlighted in red in Fig 3B. We quantified this difference in performance and Fig 3C shows the distribution of rank correlation coefficients between distances obtained with the WIP and IP metrics and the expected clustering patterns for 100 sets of 96 sequencing runs. The WIP metric outperforms the IP metric, having a significant higher mean correlation (paired Student’s T test, *n* = 84, *t* = 9.63, *df* = 83, *p* = 3.6 × 10^−15^).

### Population structure

**Fig 4:**
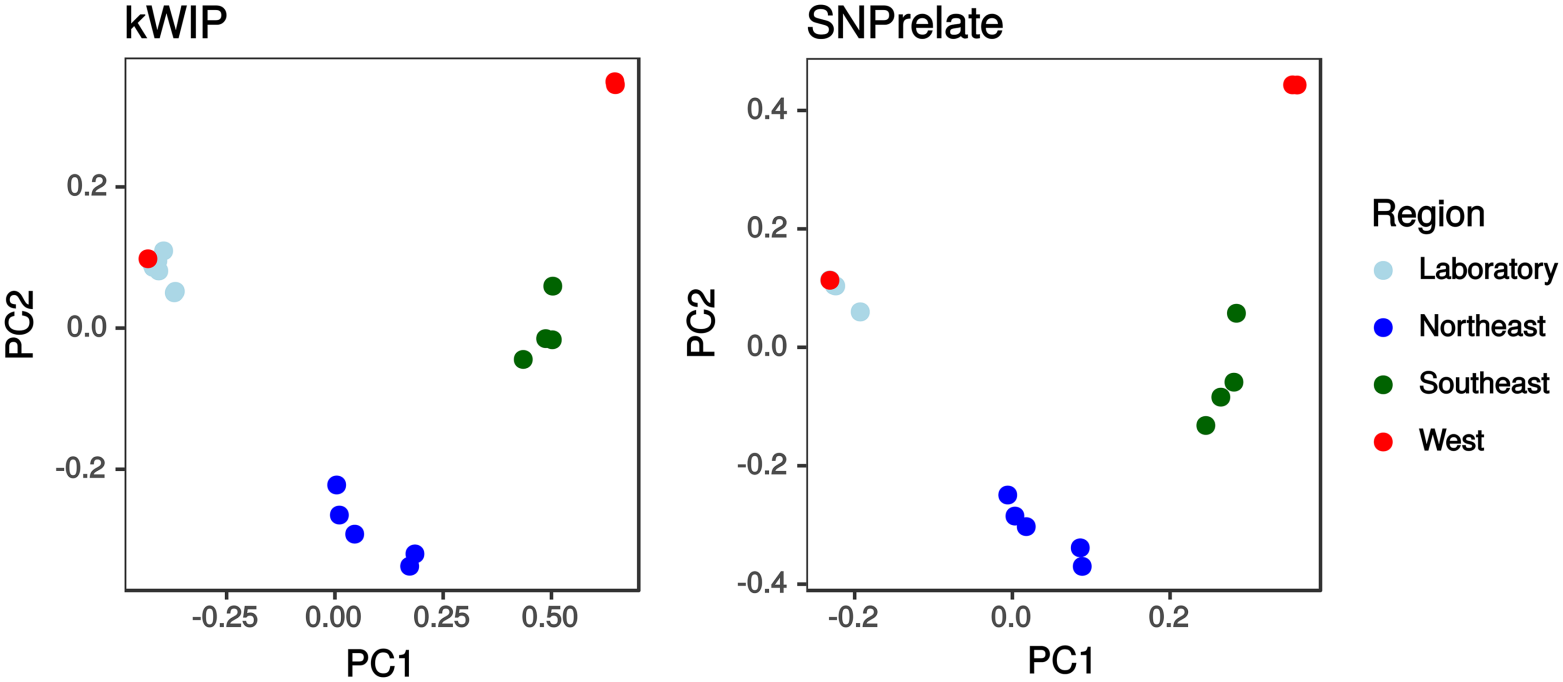
Genetic relatedness between *Chlamydomonas reinhardtii* strains based on sequencing data from [34]. SNPrelate [37] was used to compute the PCA decomposition directly from SNP genotypes provided by the authors. This replicates the analysis of [34] and is displayed on the right. On the left, we show the results of MDS performed on the distance matrix obtained with kWIP.

Flowers, et al. [34] sequenced 20 strains of *Chlamydomonas reinhardtii*; laboratory strains and wild accessions sourced from across the continental USA. By alignment- and SNP-based analysis, they find significant population structure that is mostly explained by geography [34]. In Fig 4B we display the published genetic relationships as a principal component analysis (PCA) of SNP genotypes calculated with SNPRelate [37] exactly as presented by the authors [34]. PC1 separates the laboratory strains (and one western sample) from both eastern and western samples with further structure among wild *Chlamydomonas* accession collected in western, southeastern and northeastern USA. In Fig 4A we plot the relatedness between the strains as revealed directly from the raw sequencing reads with kWIP. We note that the results are highly similar; the rank correlation between kWIP distances and genome average identity-by-state (calculated with SNPRelate [37]) is 0.95.

**Fig 5:**
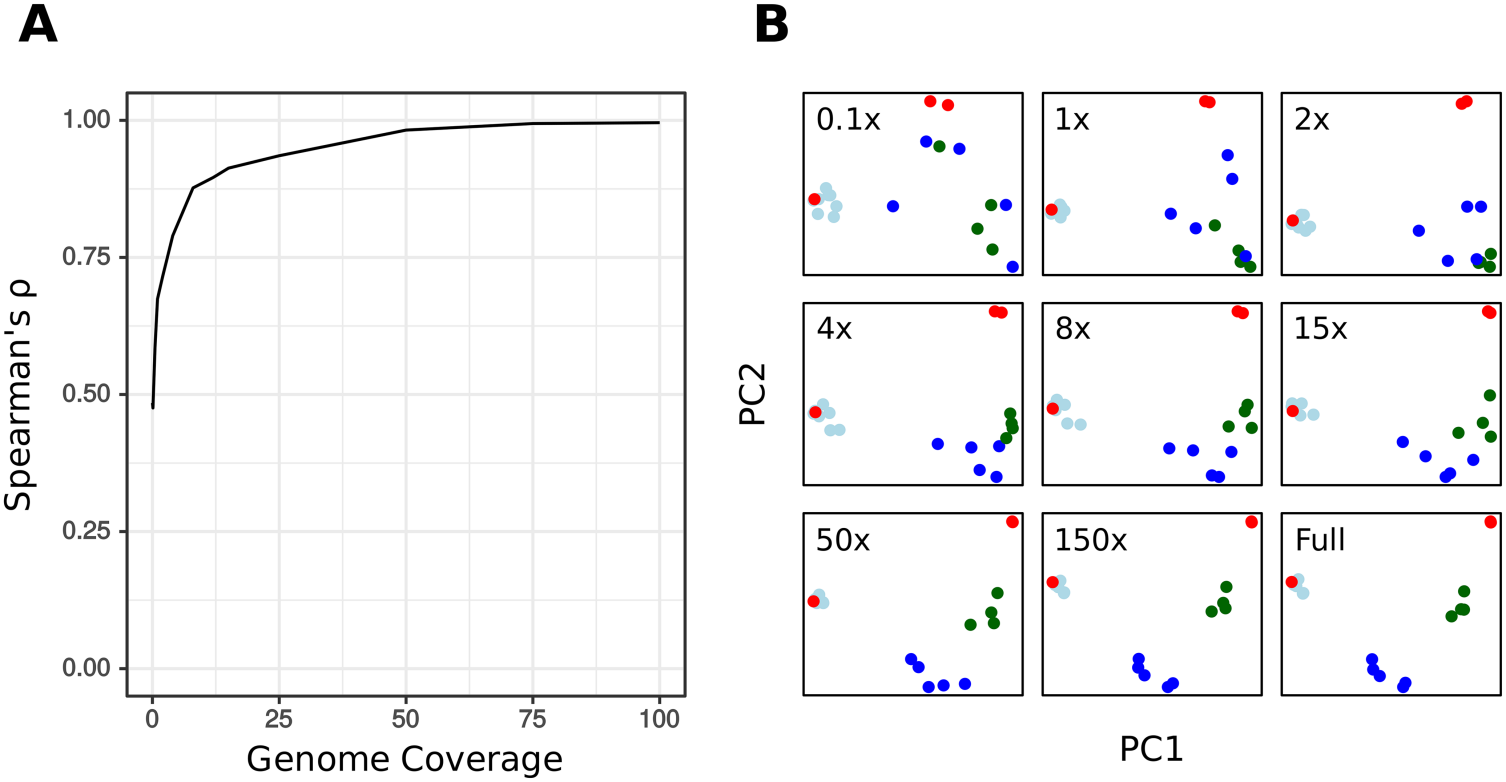
The effect of mean sequencing depth (genome coverage) on kWIP’s estimate of genetic relatedness between samples of *Chlamydomonas reinhardtii* (data from [34]). (A) Spearman’s rank correlation between sub-sampled datasets and the full dataset across a range of subset average genome coverages. (B) PCA plots of relatedness obtained using kWIP on selected sub-sampled datasets. “full” refers to the entire dataset (i.e., Fig 4), while “0.1x” refers to a sub-sampled dataset with average mean sequencing depth of 0.1 over the *C. reinhardtii* genome (likewise for 1x, 2x, and so on).

Each of the 20 strains had been sequenced to a depth of roughly 200-fold genome coverage [34]. By systematically sub-sampling this dataset we investigated the effect of coverage on the accuracy of kWIP’s similarity estimation. We find that with decreasing coverage the accuracy of the relationship estimations decreases (Fig 5A). We illustrate this decay by PCA plots of estimated genetic relatedness at varying coverages (Fig 5B). We note that the performance of kWIP to determine similarity is very good even at low coverages. A two-fold genome coverage is enough to detect the major splits in this dataset (Laboratory vs West vs East).

### Metagenome relatedness

**Fig 6:**
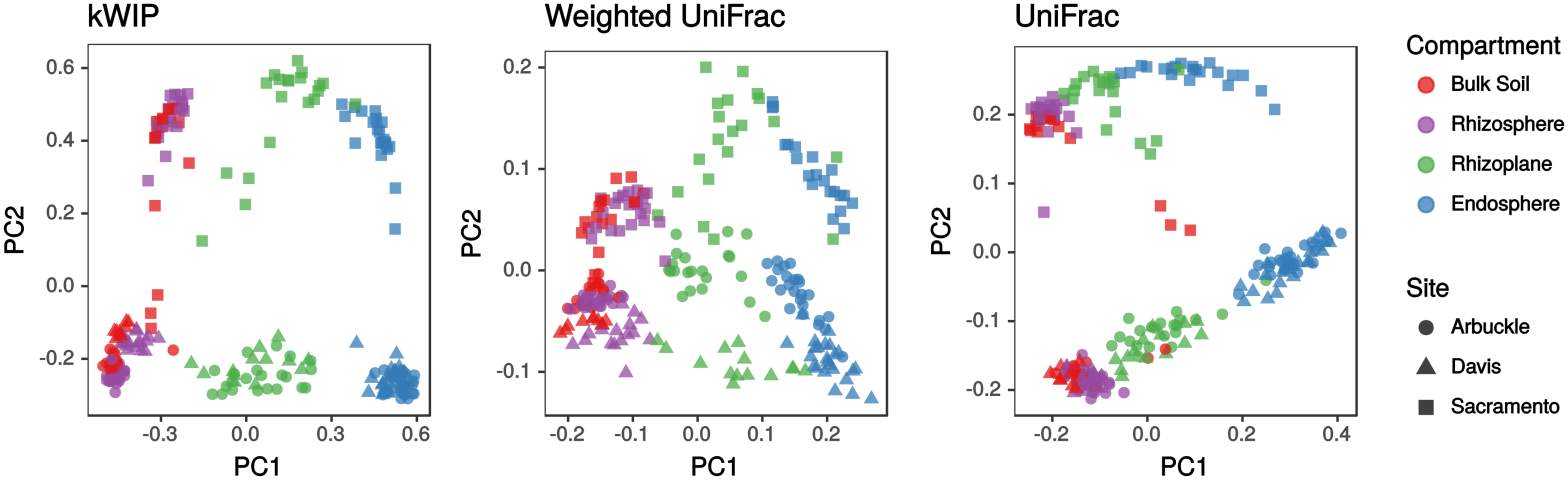
Estimation of similarity between metagenome samples. We used kWIP to examine 16S rDNA amplicon sequencing data of Edwards, *et al.* [35] and compare our kWIP result (“kWIP”) with the results as presented by Edwards, *et al.* (“Weighted UniFrac” and “UniFrac”). We find that kWIP replicates their observations of stratification of root-associated microbiomes by rhizo-compartment (PC1) and experiment site (PC2).

Edwards, *et al.* [35] sequenced 16S rDNA amplicons from rice root-associated microbiomes and find stratification of samples by rhizo-compartment, cultivation site, and cultivation practice. Analysing their raw sequencing data with kWIP, we detect highly similar stratification between microbial communities. An example is shown in Fig 6. We observe a gradient of samples from within the root, through the root-soil interface into soil, and separation by cultivation site. This replicates the separation of samples by rhizo-compartment and cultivation site published by Edwards, et al. [35], shown in Fig 6.

## Discussion

The *k*-mer Weighted Inner Product (kWIP) estimates genetic distances between samples within a population of samples directly from next generation sequencing data. kWIP does not require a reference genome sequence and is able to estimate the genetic distances between samples with less data than is typically used to call SNPs against a reference. As a *k*-mer-based method, kWIP is sequencing protocol and platform agnostic, allowing use into the future.

kWIP uses a new metric, the weighted inner product (WIP), which aims to reduce the effect of technical and biological noise and elevate the relevant genetic signal by weighting *k*-mer counts by their informational entropy across the analysis set. This weighting has the effect of down-weighting *k*-mers that are either highly abundant or present in very few samples. Those *k*-mers are typically uninformative, because they are either common, fixed, repetitive, invariable, or rare, or erroneous. By using Shannon entropy, the weights of common and infrequent *k*-mers are assigned lower, but non-zero weights, allowing them to contribute to the signal.

Euclidean distances are then calculated from these weighted inner products and kWIP outputs a matrix of pairwise distances between samples, which are easily visualised and may be used for sample classification and to cluster samples into groups. These distance matrices are amenable to quantitative comparison of genetic distance to geographic or environmental distances, for example using mantel tests or generalised dissimilarity modelling. We show high concordance between PCAs obtained using SNP data and those using kWIP. It is possible that population genetic statistics, including *F*_*ST*_, could be recovered using kWIP via a genealogical interpretation of PCA, as is proposed and shown possible for SNP datasets [38].

We have demonstrated the applicability and effectiveness of kWIP using simulations and several published datasets. Through simulations, we quantify how the novel weighting improves accuracy over both the unweighted inner product and Mash, specifically in cases where genetic differentiation or sequencing depth is low (Fig 2). With data from the 3000 rice genome dataset [33], we reconstruct known relationships between samples and sequencing runs, such as membership of samples to major genetic groups of *Oryza sativa*, and the correct clustering of replicates (Fig 3).

From sequencing reads of a population re-sequencing experiment in *Chlamydomonas* [34] we precisely recreate their visualisation of population relatedness (Fig 4). This dataset suited for comparison because Flowers, *et al.*, had based their analysis not only on variants recovered by read alignment to the reference genome, but attempted to recover and use additional variation by assembling leftover reads that did not match the reference into contigs and calling additional variants between these contigs. This approach, while reducing reference-genome bias, required extensive sequencing depth to enable de-novo assembly; the authors chose around 200-fold coverage, which in turn enabled us to assess kWIP’s performance at various sequencing depths (Fig 5).

Efficient characterisation of complex metagenome samples has traditionally relied on methods of reduced representation. We show that kWIP is able to detect structure between microbial communities based on 16S rDNA amplicon sequencing data, at least as well as current practice (Fig 6). It should be possible to apply kWIP to random shotgun sequencing data from such samples. Also, estimates of complexity and diversity within and between metagenomes are currently mostly gene based, but could also be made efficiently at the *k*-mer-level leveraging sketched data structures.

The key innovation of kWIP is the combination of a fixed-sized, probabilistic data structure (sketch) for counting *k*-mers with an entropy-weighted inner product as a measure of similarity between samples. By virtue of their fixed size, sketches enable rapid arithmetic operations on *k*-mer counts. Sketches enable kWIP to rapidly aggregate across a populations to derive weights, and to efficiently compute the inner products. These benefits outweigh the possibility of collisions between *k*-mers, which in any case have been observed to be rare [27] given appropriate sketch size. Sketching data structures are commonly used for *k*-mer counting (for example Count-Min Sketches [27, 28], and Bloom Filters [39]), but have not been widely adopted in alignment-free sequence comparison.

Weighting of inner products between sketches allows us to account for non-uniform information content of each *k*-mer. kWIP weights by Shannon entropy of presence/absence frequency across a population. This provides an assumption-free estimate of the information content of each *k*-mer. By down-weighting both rare *k*-mers introduced by rare variants or sequencing errors, as well as *k*-mers present in most or all samples, we are reducing the contribution of *k*-mers that carry less information. It is possible that other weighting functions that assume various population parameters could provide a more faithful estimate of the information content of each *k*-mer. The application of word-specific weighting has precedence in text processing, where it has been used to account for varying importance of words in a document [40]. However, because we intend kWIP to be used in situations where such parameters are either unavailable or potentially inaccurate, we prefer that our weighting is free of assumptions.

An inner product between *k*-mer counts has long been used to detect and measure sequence similarity, and is referred to as the *D*_2_ statistic. There have been many derivatives of the *D*_2_ statistic that seek to enhance its accuracy in recreating evolutionary histories (e.g., $D_{2}^{S}$, and $D_{2}^{*}$ [20–22]). kWIP does not attempt to re-create evolutionary histories, but rather estimates the similarity of genetic material as it exists today. This is sufficient and even desirable for many of kWIP’s intended uses. When validating experimental metadata, one seeks to establish whether similarity between sequencing runs matches expectations. Particularly for metagenome samples, where variation can be in both abundance and type of organisms, estimating present variation between sample genome sequences is of importance, separate to how this variation came to be.

kWIP estimates genetic similarity between sequencing runs. Because kWIP operates reference- and alignment-free, all genetic material present in the sample, the “hologenome”, will contribute to the analysis. However, we note that *k*-mers that are considered undesirable and chosen to be excluded from the analysis could easily be masked, for example by setting their weight in the weight vector to zero.

Because kWIP weights *k*-mers, and hence genome content, based on their frequency in the population being analysed, these weights change when the population changes. This allows for iterative workflows: in a first, all inclusive step the large groupings and outliers are detected; subsequently, subgroups can be analysed with increased resolution.

kWIP is purposefully designed to operate free of assumptions or prior knowledge. It is comparing data as presented in the sequencing reads without attempting to reconstruct or approximate the underlying genomes. One could think of several ways of incorporating additional knowledge, which may improve kWIP’s power to determine relatedness between underlying genomes. One could, for example, apply smoothing to the *k*-mer counts, with the goal of differentiating between *k*-mers that are genuinely not in the genomes of a sample and those that were not observed due to low coverage and/or stochastic sampling; smoothing is used in natural language modelling [41].

It is possible that alternative distance functions (e.g., Manhattan distance) over weighted sketches could improve the performance of kWIP, which currently uses Euclidean distance. Distance measures defined on presence/absence of items, such as the Jaccard index or the Jaccard index-based measures used by AAF [18] and mash [19], could also be calculated from our sketches. It may further prove valuable to explore spaced seeds [13, 16], or alternative metrics including those considering inexact matches [42, 43].

Methods that enable rapid verification of genetic resources, such as stock centre accessions or cell lines, prevent expensive and possibly catastrophic mis-identifications. Such classification tasks only require comparison with a set of reference samples rather than computing distances between all samples. Inner product kernels have been used to classify protein sequences [43, 44] and kWIP could be adapted to sample classification with tree-like structures of kernels [42] or sketches [45, 46].

Estimating the genetic relatedness between a broad collection of natural accessions provides a basis for ecological or functional studies and should be a first step towards solutions in breeding and conservation. In most population level experiments, technical sources of error are dwarfed by the error from insufficient sampling [47]. This is especially true when rare or cryptic lineages are present, and in conditions of non-random mating where population structure is substantial. Such population level noise can only be overcome by broad studies with large numbers of samples, ideally by also merging experiments [48]. When individuals from real-world populations are collected, or collated, there is normally non-uniform genetic relatedness. Initially, one seeks to group samples into more closely related families or more distantly related populations, to then develop sets for further detailed studies. Genetic outliers can represent mis-identifications and cryptic species and should be detected and excluded. *De novo* sample groupings based on whole genome relatedness also inform the selection of suitable reference individuals and/or building the necessary reference genome sequences. The initial characterisation process must avoid biases and have minimal per sample cost. The use of kWIP allows one to base the analysis of diversity among samples on low coverage, whole-genome sequence data and thus facilitates large, balanced study designs. More broadly, experiments are condemned to be inconclusive and irreproducible if samples are somehow mislabelled or misidentified. An initial step in all analyses of genetic or functional variation must involve the verification of sample identity [6]. This preliminary analysis should preferably use whole-genome sequence data, be *de novo*, unbiased, and agnostic to sequencing protocol and technology. kWIP is an efficient implementation of such a tool.

### Availability and future directions

kWIP is implemented in C++ and licensed under the GNU GPL. Source code and pre-compiled executables are available from https://github.com/kdmurray91/kwip. Documentation and tutorials are available from https://kwip.readthedocs.io. Docker images, Snakemake workflows and Jupyter notebooks used to perform all analyses presented here are available online at https://github.com/kdmurray91/kwip-experiments; the respective software versions are noted within the repository. When given a population of samples, kWIP performs all pairwise comparisons, which scales quadratically with regards to the number of samples ($O(n^{2})$), but parallelises pairwise similarity calculations across cores of a multi-threaded computer to ensure fast operation. Analyses of very large data sets, i.e., beyond 10,000s of samples, will benefit from further optimisation to the implementation of kWIP, including parallelisation across distributed memory systems with MPI. For each pairwise comparison, the two sketches and the weight vector must fit in main memory. This limits the size of the sketches and the number of pairwise comparisons that will run efficiently in parallel on a given node.

## Materials and methods

We demonstrate kWIP’s performance with both real and simulated datasets. With simulations we quantify the performance of kWIP. To demonstrate the utility of kWIP in real-world, low-coverage, large-scale population genomics datasets, we analyse data from the 3000 Rice Genomes Project [32, 33]. To show that kWIP estimates genetic similarity as well as current best practice SNP-based methods, we re-analysed a population genomics study on 20 strains of *Chlamydomonas reinhardtii* [34] with kWIP and compare our result to the published results. Lastly, using data from a study on root-associated microbiomes of rice [35], we show that kWIP is able to separate microbial communities from 16S rDNA amplicon data at least as well as current best-practice methods in metagenomics.

We provide all information necessary to reproduce our work: the kWIP analyses performed here are implemented in Snakemake workflows [49], which describe all steps and software parameters; random seeds have been fixed where necessary. All downstream analyses are available as Jupyter notebooks [50, 51]. Both the Snakemake workflows and Jupyter notebooks are available online at https://github.com/kdmurray91/kwip-experiments; the respective software versions are noted within this repository.

### Simulations

We simulated several datasets to empirically quantify the performance of kWIP. Twenty populations with 12 individuals each were simulated using scrm [52]. Branch lengths within each population were normalised such that the mean pairwise genetic distance (*π*) was equal. Branch lengths were then scaled over a range of *π* (between 0.001 and 0.2) to test the effect of mean pairwise genetic distance on accuracy. Genome sequences of 1 Mbp genomes were simulated with DAWG2 [53] and from those short read data for three replicate sequencing runs per individual were generated at various mean coverages (between 1- and 128-fold) using Mason2 [54]. We attempted to emulate the reality of sequencing experiments by introducing random variation in read numbers between replicate runs (coefficient of variation of 0.3). These simulated sequencing runs were then used to estimate genetic similarity with kWIP and mash [19]. For analysis with kWIP we used khmer to hash *k*-mers of length 20 into sketches with 10^7^ bins. We estimated genetic similarity with kWIP, using the weighted (“WIP”) and unweighted (“IP”) metrics. On the same data we performed two analyses with Mash, counting 20-mers into sketches of size 10^4^. For one analysis, we invoked the abundance filter within mash sketch such that only *k*-mers observed at least twice were considered (“Mash (AF)”), whereas the other analysis considered all *k*-mers regardless of abundance (“Mash”).

The performance of our metrics was measured relative to the true pairwise distances between the simulated samples. The true distance matrix between samples was calculated from the simulated, aligned sample genomes (which DAWG2 produces) with scikit-bio. Sample-wise distances were replicated three times to allow comparison to the distances obtained from the three simulated sequencing runs. Performance was calculated as Spearman’s rank correlation (*ρ*) between all pairwise distances using scipy [55].

### Datasets

With several published datasets we demonstrate the performance and utility of kWIP in real-world scenarios. In all cases, sequence data files for sequencing runs were obtained from the NCBI Short Read Archive using sra-py [56]. Reads were extracted using the SRA toolkit to FASTQ files. Low base quality regions were removed using sickle [57] in single-end mode. Counting of *k*-mers into count files (sketches) was performed using the load-into-counting.py script of khmer. Genetic similarity was estimated using kWIP, with the WIP and IP metrics.

To assess how well kWIP recovers replicate samples and known sample hierarchies at low sequencing coverage, we turned to publicly available sequence data from the 3000 Rice Genomes project [32, 33]. Samples of the 3000 Rice Genomes project had been sequenced on the Illumina HiSeq2000 platform with technical replicates of individual sequencing libraries split between 6 or more sequencing lanes [32, 33]. Furthermore, there is a rather strong subdivision of rice (*Oryza sativa*) into subgroups. We compiled 100 sets of 96 runs, i.e., for each set we chose 16 samples with 6 replicate runs. We ensured that 8 samples each were described by [32] as belonging to the Indica and Japonica subgroups of *O. sativa*. We estimated the genetic similarity between runs in each of these 100 sets with kWIP. The true distances between the different runs in the 3000 rice datasets are not known, but a topology and sample hierarchy can be inferred from the metadata. We hence assessed the performance of kWIP in accurately clustering replicates and recovering population structure against a mock distance matrix that reflects the expected topology. We created a distance matrix in which each run had a distance of zero to itself, a distance of 1 to each of its technical replicates (i.e., the other sequencing runs belonging to the same sample), a distance of 2 to each run from other samples in the same rice group (Indica or Japonica), and a distance of 4 to each run from a sample belonging to the respective other rice group. We then used scipy to calculate Spearman’s rank correlation between this mock matrix and each distance matrix obtained from real data using kWIP. A paired Student’s t-test was performed between the estimates of relatedness from the WIP and IP metrics with the t.test function in R. We used hierarchical clustering to visualise these relationships, performed in R with the hclust function.

We use whole genome sequencing data on 20 strains of *Chlamydomonas reinhardtii* [34] to demonstrate that kWIP is able to detect population structure in a real-world dataset and to examine the effect of sample sequencing depth (coverage) on accuracy of kWIP. Genetic relatedness between the 20 *Chlamydomonas reinhardtii* samples from this study was estimated with kWIP using the WIP metric. Classic Multi-dimensional Scaling (MDS) of the kWIP distance matrix was performed using the cmdscale function in R. For Euclidean distance matrices, MDS is equivalent to PCA [58]. We compare our MDS results with the principal component analysis (PCA) decomposition of SNP genotypes calculated with function snpgdsPCA in SNPrelate [37], working from a VCF file provided by Flowers *et al.* [34]. From the aforementioned SNP data we calculated genome-wide average identity-by-state (IBS) with the snpgdsIBS function in SNPrelate [37]. Rank correlation between kWIP distances and 1-IBS was calculated with function cor in R [59].

We examined the effect of mean sequencing depth (coverage) on the accuracy of kWIP by random sub-sampling from the sequencing data of each sample. We sub-sampled to coverages of between 0.01- and 200-fold average genome coverage (0.01, 0.1, 0.5, 1, 2, 4, 8, 12, 15, 25, 50, 75, 100, 150, 200x) across samples using the sample command of seqtk [60]. We attempted to preserve the coefficient of variation in read numbers that existed in the original dataset (0.12) by sampling a random number of reads from the appropriate normal distribution. Spearman’s rank correlation (*ρ*) was used to compare pairwise distances calculated at each sub-sampled coverage to those from the original dataset with function cor in R [59].

To demonstrate that kWIP can determine the relatedness of samples in a typical metagenomic dataset, we used next generation sequencing data from a study on rice root associated microbiomes [35] representing 16S rDNA amplicons from soil and root samples. Relatedness between samples was estimated using kWIP with the WIP metric, and MDS was performed as above.
